# Co-Introduced Functional CCR2 Potentiates In Vivo Anti-Lung Cancer Functionality Mediated by T Cells Double Gene-Modified to Express WT1-Specific T-Cell Receptor

**DOI:** 10.1371/journal.pone.0056820

**Published:** 2013-02-18

**Authors:** Hiroaki Asai, Hiroshi Fujiwara, Jun An, Toshiki Ochi, Yukihiro Miyazaki, Kozo Nagai, Sachiko Okamoto, Junichi Mineno, Kiyotaka Kuzushima, Hiroshi Shiku, Hirofumi Inoue, Masaki Yasukawa

**Affiliations:** 1 Department of Bioregulatory Medicine, Ehime University Graduate School of Medicine, Ehime, Japan; 2 Department of Cell Growth and Tumor Regulation, Ehime University Proteo-Medicine Research Center, Ehime, Japan; 3 Princess Margaret Hospital, Ontario Cancer Institute, Ontario, Canada; 4 Department of Pediatrics, Ehime University Graduate School of Medicine, Ehime, Japan; 5 Center for Cell and Gene Therapy, Takara Bio Inc., Shiga, Japan; 6 Division of Immunology, Aichi Cancer Center, Aichi, Japan; 7 Department of Cancer Vaccine and Immuno-Gene Therapy, Mie University Graduate School of Medicine, Mie, Japan; 8 Department of Biochemistry and Molecular Genetics, Ehime University Graduate School of Medicine, Ehime, Japan; University of Pittsburgh, United States of America

## Abstract

**Background and Purpose:**

Although gene-modification of T cells to express tumor-related antigen-specific T-cell receptor (TCR) or chimeric antigen receptor (CAR) has clinically proved promise, there still remains room to improve the clinical efficacy of re-directed T-cell based antitumor adoptive therapy. In order to achieve more objective clinical responses using ex vivo-expanded tumor-responsive T cells, the infused T cells need to show adequate localized infiltration into the tumor.

**Methodology/Principal Findings:**

Human lung cancer cells variously express a tumor antigen, Wilms' Tumor gene product 1 (WT1), and an inflammatory chemokine, CCL2. However, CCR2, the relevant receptor for CCL2, is rarely expressed on activated T-lymphocytes. A HLA-A2402^+^ human lung cancer cell line, LK79, which expresses high amounts of both *CCL2* and *WT1* mRNA, was employed as a target. Normal CD8^+^ T cells were retrovirally gene-modified to express both CCR2 and HLA-A*2402-restricted and WT1_235–243_ nonapeptide-specific TCR as an effector. Anti-tumor functionality mediated by these effector cells against LK79 cells was assessed both in vitro and in vivo. Finally the impact of CCL2 on WT1 epitope-responsive TCR signaling mediated by the effector cells was studied. Introduced CCR2 was functionally validated using gene-modified Jurkat cells and human CD3^+^ T cells both in vitro and in vivo. Double gene-modified CD3^+^ T cells successfully demonstrated both CCL2-tropic tumor trafficking and cytocidal reactivity against LK79 cells in vitro and in vivo. CCL2 augmented the WT1 epitope-responsive TCR signaling shown by relevant luciferase production in double gene-modified Jurkat/MA cells to express luciferase and WT1-specific TCR, and CCL2 also dose-dependently augmented WT1 epitope-responsive IFN-γ production and CD107a expression mediated by these double gene-modifiedCD3^+^ T cells.

**Conclusion/Significance:**

Introduction of the CCL2/CCR2 axis successfully potentiated in vivo anti-lung cancer reactivity mediated by CD8^+^ T cells double gene-modified to express WT1-specific TCR and CCR2 not only via CCL2-tropic tumor trafficking, but also CCL2-enhanced WT1-responsiveness.

## Introduction

Despite recent therapeutic progress, the overall survival of patients with advanced lung cancer still remains poor [1], and therefore the exploration of new therapies remains a desirable objective. Results from clinical trials of anti-tumor adoptive therapy using ex vivo-expanded tumor-responsive T cells, mainly tumor-infiltrating T lymphocytes (TIL), for the treatment of advanced melanoma have demonstrated an impressive clinical responsiveness. On the other hand, there are certain drawbacks, such as the complexity of the procedures and the difficulty in maintaining the therapeutic quality of long-term-cultured T cells [2]. Recent technical advances involving gene modifications to introduce tumor-responsive receptors into therapeutic T cells – such as the tumor antigen-specific T-cell receptor (TCR) and chimeric antigen receptor (CAR) – have largely overcome these drawbacks [3]–[5]. However, as the range of suitably responsive tumors is still limited, we have proposed some new options, such as HLA-A*2402-restricted WT1-specific TCR [6] and HLA-A*0201-restricted Aurora kinase A (AURKA)-specific TCR [7], for the treatment of human leukemias. Another technical advance we have proposed is a novel TCR vector system which simultaneously delivers shRNAs for endogenous TCR α/β genes (siTCR vector) [8], thus reducing the formation of mispaired TCR, the potential risk of lethal acute GVHD [9].

WT1 is a well-known tumor antigen expressed to various degrees by human lung cancer cells [10], and WT1 expression has been shown clinically to have prognostic value in lung cancer patients [11]. Using a xenografted mouse model, we have previously explored the anti-lung cancer therapeutic potential of an ex vivo-expanded clonal cytotoxic T cell line (CTL) [12], TAK-1, which specifically recognizes the WT1_235–243_ nonamer epitope in the context of HLA-A*2402 [13].

On the other hand, insufficient infiltration of therapeutic T cells into localized tumor sites is a constraint for successful treatment [14]. In order to augment the tumor trafficking activity of infused therapeutic T cells, their responsiveness to appropriate chemokines produced by the tumor cells or tumor-infiltrated immune cells is required. First by Kershaw et al. [15], a series of preclinical studies based on this concept have been conducted [16]–[19]. However, the principal issue of which chemokine-chemokine receptor pair should be chosen for clinical application still remains to be settled. In the present study, in order to examine the potential advantages of co-introduction of a chemokine-chemokine receptor axis for antitumor adoptive immunotherapy, we employed as a model genetically redirected T cells targeting WT1 for the treatment of human lung cancer.

In this study, we found that CC chemokine 2 (CCL2) was produced to variable degrees by human lung cancer cell lines, and that LK79, a HLA-A*2402^+^ small-cell lung cancer (SCLC) cell line overexpressing *WT1* mRNA, produced extremely high amounts of CCL2. LK79 was killed by CD8^+^ T cells gene-modified to express the WT1-specific TCR originating from TAK-1. On the other hand, CCR2, the specific receptor for CCL2, was hardly expressed on these transfectants. Taken together, the data suggested that in order to demonstrate our proof-of-concept, it would be sensible to employ the CCR2-CCL2 axis in the setting of redirected T cells targeting WT1 and lung cancer. Because treatment of SCLC still remains challenging [20], we considered that the use of LK79, a SCLC cell line, as a target, might open a new avenue of therapy for SCLC.

In the present study, we examined in detail the anti-lung cancer functionality mediated by double-transfected CD8^+^ T cells to express WT1-specific TCR and CCR2 against LK79 cells, both in vitro and in vivo. Based on our observations, we discussed the clinical feasibility of this strategy for adoptive immunotherapy against human lung cancer.

## Materials and Methods

### 1. Ethics Statement

Approval for this study was obtained from the Institutional Review Board of Ehime University Hospital. Written informed consent was given by all healthy volunteers in accordance with the Declaration of Helsinki. All in vivo mouse experiments were approved by the Ehime University animal care committee.

### 2. Cells

Jurkat cells (ATCC) and human lung cancer cell lines positive for either HLA-A*2402^+^, WT1 mRNA, or both were employed. The latter included LC99A (large cell carcinoma origin) [21], LK79 (small cell carcinoma) [21], RERF-LC-A1 (squamous cell carcinoma) [21] and LC11-18 (adenocarcinoma) [22]. PC-9 (adenocarcinoma) [21] was positive for HLA-A*2402^+^ but negative for WT1 mRNA, Sq-1 (squamous cell carcinoma) [21], LC65A (small cell carcinoma) [21], QG56 (squamous cell carcinoma) [21], LK87 (adenocarcinoma) [21], and 1–87 (adenocarcinoma) [21] were negative for HLA-A*2402. All of these previously published lung cancer cell lines were kindly provided by Dr. Akio Hiraki (Okayama University Graduate School, Okayama, Japan), and cultured as reported previously [12]. The Jurkat/MA cell line (kindly provided by Prof. Erik Hooijberg, Vrije Universiteit Medisch Centrum, Amsterdam, The Netherlands) is a Jurkat cell subclone previously established by Prof. Erik Hooijberg and colleagues that lacks endogenous TCR expression and stably expresses both the human CD8α gene (*hCD8α*) and an *NFAT-luciferase* gene construct for detection of signaling via newly introduced TCRs [23]. The HLA-A*2402 gene-transduced C1R cell line (C1R-A24) was also cultured in RPMI1640 medium supplemented with 10% fetal calf serum and 0.5 mg/mL hygromycin B (Invitrogen). B-lymphoblastoid cell lines (B-LCLs) were established by transformation of peripheral blood B lymphocytes with Epstein-Barr virus. Peripheral blood mononuclear cells (PBMCs) from healthy volunteers were isolated and stored in liquid nitrogen until use.

### 3. Mice

Six-week-old NOD/scid/γc^null^ (NOG) female mice were purchased from the Central Institute for Experimental Animals [24] and maintained in the institutional animal facility at Ehime University.

### 4. Flow cytometry

Surface markers of transfectants or non-gene-modified T cells were labeled with anti-CD8, anti-CD4, anti-CD3 and anti-CD45 mAbs (BD Biosciences), anti-CD25 and anti-CD69 mAbs (BioLegend), anti-Vβ5.1 mAb (Beckman Coulter), anti-CCR2 mAb (R&D Systems), and HLA-A*2402/WT1_235–243_ tetramer-PE or HLA-A*2402/HIV-1 Env_584–592_ tetramer-PE, as a negative control. Flow cytometry was conducted using a Gallios flow cytometer (Coulter), and data analysis was performed using Flow Jo Version 7.2.2 software (TreeStar).

### 5. WT1-siTCR retroviral vector and CCR2 retroviral vector

The HLA-A*2402-restricted and WT1_235–243_-specific TCR-α (Vα20/J33/Cα) and TCR-β (Vβ5.1/J2.1/Cβ2) genes, which originated from TAK-1 [13], were cloned into our novel retroviral siTCR vector (WT1-siTCR vector), then transduced into T cells using this vector as described previously [6], [8]. The full-length cDNA of the human *CCR2* gene (1083 bp) (NM_001123396.1) was cloned and codon-optimized (GeneArt), then inserted into the pRetroX-IRES-DsRed Express vector (Clontech). Ecotropic retroviral vectors were obtained by transient co-transfection with other components (Takara Bio) into the HEK293 cell line; subsequently, GaLV-pseudotyped retroviral vectors were obtained by sequential transfection of these vectors into the PG13 cell line (Takara Bio).

### 6. Transduction of the *TCR* and *CCR2* genes

Jurkat/MA cells and healthy donor T cells were gene-modified to express WT1-specific TCR and CCR2 as described previously [6]. Briefly, on day 1, 1×10^6^ T cells per well in GT-T503 (Takara Bio) with 5% human serum, 0.2% human albumin, 50 U/mL recombinant human IL-2 (R&D Systems), 10 ng/mL recombinant human IL-15 (PeproTec Inc.), and 100 ng/mL recombinant human IL-21 (Shenandoah Biotechnology Inc.) were added to a 24-well culture plate pretreated with anti-human CD3 mAb (BioLegend). Jurkat/MA cells were cultured in IMDM with 8% FCS and 50 mg/mL hygromycin B. On day 3, cultured T cells or Jurkat/MA cells were transferred to a retrovirus-preloaded RetroNectin-coated 24-well plate, centrifuged at 2000×*g* for 2 hours and rinsed with PBS. Cells were then applied again to another similarly pre-treated 24-well plate for the second transduction. The WT1-si*TCR-* and *CCR2* -transduced T cells were stimulated weekly with mitomycin-C (MMC) (Kyowa Hakko)-treated and heteroclitic WT1_235–243_ peptide (CYTWNQMNL)-pulsed HLA-A*2402-positive LCLs. In some experiments, CD8^+^ T cells transfected to express WT1-specific TCR were isolated using anti-Vβ5.1-FITC mAb (Beckman Coulter) and anti-FITC-conjugated immunomagnetic beads (Miltenyi Biotec), and *CCR2* transfectants were also isolated using anti-CCR2-PE mAb (R&D Systems) and anti-PE-conjugated immunomagnetic beads (Miltenyi Biotec).

### 7. Assessment of chemokines produced by human lung cancer cell lines

All primers for quantitative real-time PCR (qRT-PCR) used for assessment of 12 selected chemokines produced by 10 human lung cancer cell lines are listed in Table 1. Briefly, total RNA was extracted from each cell line with an RNeasy Mini Kit (Qiagen) and cDNA was synthesized. qRT-PCR for chemokine mRNA was performed using a QuantiTect SYBER Green PCR kit (Qiagen) as described previously [7]. Human hypoxanthine phosphoribosyltransferase 1 (*hHPRT1*) mRNA (NM_000194) was used as an internal control. The PCR conditions were 50°C for 2 min, 95°C for 15 min, 50 cycles of 95°C for 15 s, 60°C for 1 min, 95°C for 15 s and then 60°C for 1 min. These samples were analyzed using an ABI prism 7500 Sequence Detection System (Applied Biosystems). The expression of each chemokine mRNA was corrected by reference to that of *hHPRT1* mRNA, and the relative amount of each chemokine mRNA was calculated by the comparative ΔC_t_ method. CCL2 protein produced by each cell line was assessed using an ELISA kit (R&D Systems). Streptavidin-HRP was used for color development, and luminointensity was measured using IMMUNO-MINI (NJ-2300; Microtec).

**Table 1:** Assessment of 12 selected chemokines.

	GenBank Accession No.	Forward primer	Reverse primer
CCL2	NM_002982.3	GCTCATAGCAGCCACCTTCATTC	GGACACTTGCTGCTGGTGATTC
CCL3	NM_002983.2	CCTGCTCAGAATCATGCAGGTC	AGCACTGGCTGCTCGTCTCA
CCL4	NM_002984	CTAGTAGCTGCCTTCTGCTCTCCAG	AATCTACCACAAAGTTGCGAGGAAG
CCL5	NM_002985.2	ACCAGTGGCAAGTGCTCCAAC	CTCCCAAGCTAGGACAAGAGCAAG
CCL8	NM_005623	TGCTCATGGCAGCCACTTTC	CACGTTAAAGCAGCAGGTGATTG
CCL22	NM_002990.3	GCGTGGTGAAACACTTCTACTGGA	TCATCTTCACCCAGGGCACTC
CXCL9	NM_002416.1	AGGGTCGCTGTTCCTGCATC	TTCACATCTGCTGAATCTGGGTTTA
CXCL10	NM_001565.3	GGCCATCAAGAATTTACTGAAAGCA	TCTGTGTGGTCCATCCTTGGA
CXCL11	NM_005409	TGAAGTAGCAGCAACAGCACCA	CCAGGGCCTATGCAAAGACAG
CXCL12	NM_199168.2	GAGCCAACGTCAAGCATCTCAA	TTTAGCTTCGGGTCAATGCACA
CXCL16	NM_022059	TGTGGCACCTGACTCTAATACCTGA	TTCCATAACAGCCTGGGCAAC
CX3CL1	NM_002996.3	TGCCATCTGACTGTCCTGCTG	CATCTTGCTGCACGTGATGTTG
hHPRT1	NM_000194.2	GGCAGTATAATCCAAAGATGGTCAA	GTCAAGGGCATATCCTACAACAAAC

hHPRT1 indicates “Homosapiens Hypxanthine Phosphoribosyltransferase 1”.

### 8. WT1-responsive luciferase production mediated by double-transfected Jurkat/MA cells

To measure the impact of CCL2 ligation to co-introduced CCR2 on WT1 epitope-responsive TCR signaling, the Jurkat/MA cell line, which is devoid of endogenous TCR, and stably expresses *hCD8α* and an *NFAT-luciferase* reporter gene (Jurkat/MA/CD8α/luc) was employed. Briefly, the WT1-si*TCR* and *CCR2* genes were retrovirally transduced into Jurkat/MA/CD8α/luc cells as described previously [7]. Double gene-modified Jurkat/MA/CD8α/luc cells, double positive for Vβ5.1 and CCR2, were isolated, expanded and subjected to functional analysis. Two million double-transfected Jurkat/MA/CD8α/luc cells were co-incubated with 1×10^6^ MMC-treated C1R-A24 cells with or without loaded WT1 peptide (20 µM) as a stimulator in various concentrations of human recombinant CCL2 (Peprotech) for 12 hours at an effector∶target ratio of 2∶1. Single-transfected Jurkat/MA/CD8α/luc cells solely expressing WT1-specific TCR were used as a control. Subsequently these cells were lysed and subjected to luciferase assay using a PicaGene-Dual-SeaPansy Kit (TOYO inki). Luciferase activity was measured using a Lumicounter 700 (Microtec Nition).

### 9. ^51^Cr-release assay

To determine the cytotoxic activity mediated by *WT1-siTCR* gene-transduced CD8^+^ T cells, standard ^51^Cr-release assays were performed as described previously [25]. Briefly, 10^4^ unpulsed or peptide-pulsed target cells were labeled with ^51^Cr (Na_2_CrO_4_: MP Bio Japan) and incubated at various ratios with effector cells in 200 µL of culture medium in 96-well round-bottomed plates. For inhibition assay, cells were cultured in the presence of either an anti-HLA class I framework mAb (w6/32; ATCC) or a control anti-HLA-DR mAb (L243; ATCC). After 5 hours of incubation with effector cells, 100 µL of supernatant was collected from each well. The percentage of specific lysis was calculated as: (experimental release cpm-spontaneous release cpm)/(maximal release cpm-spontaneous release cpm)×100 (%).

### 10. IFN-γ secretion assay

Five hundred thousand double-transfected normal peripheral CD8^+^ T cells expressing both WT1-specific TCR and CCR2 were co-incubated with 1×10^5^ WT1 peptide-pulsed (1 µM) or unpulsed C1R-A24 cells for 24 hours in various concentrations of CCL2. IFN-γ in the culture supernatant was similarly measured using an ELISA kit (R&D Systems).

### 11. CD107a assay

CD107a expression mediated by double-transfected CD8^+^ T cells in response to stimulation with WT1 peptide was examined as described previously [26]. Briefly, 1×10^5^ C1R-A24 cells were seeded into a 96-well round-bottom plate and incubated with or without WT1 peptide for 2 hours in various concentrations of CCL2. Then, 2×10^5^ of these double-transfected CD8^+^ T cells were seeded into each well with FITC-conjugated CD107a mAb (BioLegend), and incubated for 3 hours. The cells were then additionally labeled with anti-CD3, anti-CD8 mAbs (BD Biosciences) and PE-conjugated HLA-A*2402/WT1_235–243_ tetramer, and subjected to flow cytometric analysis.

### 12. Transwell experiments

Functional validation of transduced CCR2 was conducted in vitro employing transwell experiments. In brief, 2×10^5^/well *CCR2*-transfected Jurkat cells were placed in the upper well, and 500 µL RPMI 1640 with 10% FCS culture medium containing various concentrations of CCL2 was added to the bottom well of a 3-µm pore-size 24-well transwell plate (Coster). After 4 hours of incubation, the cells in the bottom well were counted using the trypan blue dye exclusion method. All experiments were conducted in triplicate and independently three times. When human lung cancer cell lines were seeded into the bottom wells, transwell experiments were similarly conducted after a 24-hour pre-incubation. A blocking experiment was conducted using ant-CCR2 mAb (R&D Systems). Finally, both tumor trafficking and cytocidal activities mediated by double gene-modified CD8^+^ T cells were examined in the same transwell experiment. Briefly, using a 3-µm pore-size 6-well transwell plate, LK79 cells were seeded into the bottom well at 10^6^/well and incubated for 24 hours. Then, double-transfected CD8^+^ T cells were loaded onto the upper well at 2×10^6^/well. WT1-specific *TCR* single-transfected CD8^+^ T cells were used as a control. After 12 hours of incubation, each supernatant in the bottom well was harvested. After centrifugation at 1200 rpm for 5 min for removal of residual cells, 100 µL of each supernatant was subjected to ELISA assay (Roche) for lactate dehydrogenase (LDH) having leaked from the LK79 cells disrupted by migrated WT1-specific effector T cells. Experiments were conducted in triplicate. Since the retroviral *CCR2* gene expression vector simultaneously delivered a red-colored fluorescent protein (Ds-Red), double-transfected effector cells having migrated to the bottom well were also detected by confocal microscopy (A-1R, Nikon, Japan).

### 13. In vivo WT1 antigen-independent migration

In a xenografted mouse model, CCL2-dependent tumor trafficking activity mediated by *CCR2* and *luciferase* double-transfected normal CD3^+^ T cells was assessed using bioluminescence imaging assay. Briefly, 1×10^7^ LK79 cells were subcutaneously inoculated into the abdominal wall of 1 Gy-irradiated 9-week-old NOG mice (n = 6). One cohort (n = 3) received intravenous administration of single *luciferase*-transfected human CD3^+^ T cells (5×10^6^ cells/mouse) as a control, and the other cohort (n = 3) received double-transfected CD3^+^ T cells on day 0. Thereafter all mice received serial intraperitoneal administrations of 500 IU recombinant human IL-2 (Roche) on days 0, 2, 4, and 6. Serial acquisition of luciferase photon counts using luciferin (Promega Corporation) was carried out on days 1, 3, 5, and 7 using AEQUORIA (Hamamatsu Photonics, Japan), and analyzed using AQUACOSMOS software (Hamamatsu Photonics).

### 14. In vivo tumor-suppressive activity mediated by infused double-transfected effector T cells in a therapeutic mouse model

For therapeutic adoptive transfer experiments, we prepared *luciferase*-transfected LK79 cells (LK79/luc) whose CCL2 production was equal to that of the parental LK79 cells. All 9-week-old NOG mice (n = 18) were 1 Gy-irradiated and then inoculated subcutaneously with 5×10^6^ LK79/luc cells into the abdominal wall on day 0. These mice were divided into three cohorts: i) those intravenously administered 5×10^6^ OKT-3/IL-2-activated CD8^+^ T cells without any gene modification (n = 6), ii) those treated with 5×10^6^ single WT1-specific *TCR*-transfected CD8^+^ T cells from an identical donor (n = 6), and iii) those treated with 5×10^6^ double-transfected CD8^+^ T cells also from the same donor (n = 6). The mice in each cohort received the same cell therapy weekly three times in total (on days 4, 11, and 18). Serial acquisition of luciferase photon counts for inoculated LK79/luc cells was carried out. The photon counts relative to that on the first day of cell therapy, which indicated the residual tumor mass burden, were calculated in each mouse.

### 15. Statistical analysis

The paired *t* test was used to assess differences between two groups, and a one-way factorial analysis of variance followed by a Tukey's post-hoc test was used for comparisons among three groups; differences at *p*<.05 were considered significant.

## Results

### 1. Production of various amounts of CCL2 by human lung cancer cell lines, and rare expression of the corresponding receptor CCR2 on T cells activated using OKT-3 and IL-2

**Figure 1:**
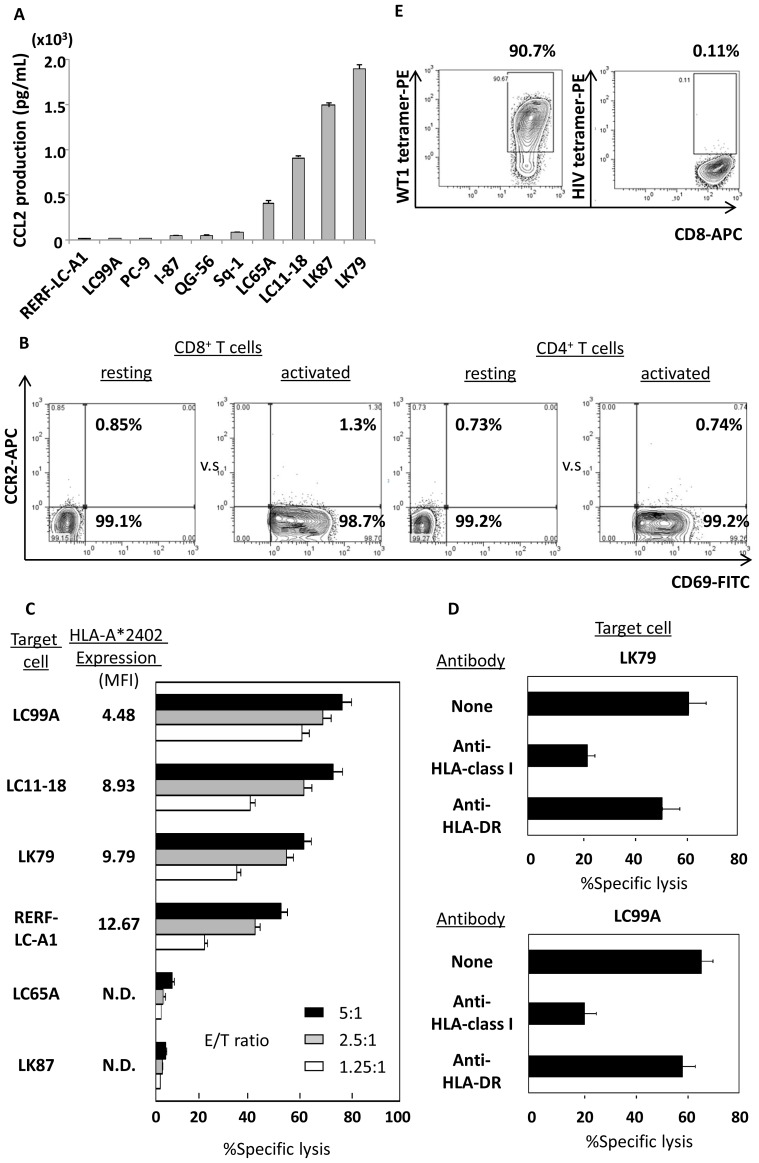
Human lung cancer cells produce variable amounts of CCL2 and show sensitivity to cytocidal activity mediated by CD8^+^ T cells genetically engineered to express WT1-specific TCR. (A) ELISA assay revealed that 10 human lung cancer cell lines examined produced various amounts of CCL2 in the culture supernatant. Error bars represent SDs. (B) Similarly to transduction of the WT1-specific *TCR* gene, CD69-positive CD8^+^ and CD4^+^ T cells activated using IL-2 and OKT-3 rarely displayed cell-surface CCR2. A representative example of 5 cases is shown. (C) CD8^+^ T cells gene-modified to express HLA-A*2402-restricted and WT1_235–243_ nonamer-specific TCR successfully displayed cytocidal activity against lung cancer cell line cells in a HLA-A*2402-restricted manner, as determined by standard ^51^Cr release assay. Error bars represent SDs. MFI indicates mean fluorescence intensity, N.D indicates less than detectable. (D) Such anti-lung cancer activity (vs. LK79 and LC99A) was inhibited by anti-HLA class I framework mAb (clone w6/32), but not by anti-HLA-DR mAb (clone L243), again illustrating that the introduced WT1-specific TCR-mediated tumoricidal activity was HLA-class I-restricted. Error bars represent SDs. (E) Tetramer assay showed that effector cells used in these experiments were positive for WT1/HLA-A*2402-tetramer. HIV/HLA-A*2402 tetramer was employed as a negative control.

The expression profiles of the 12 chosen chemokine mRNAs produced by each of the human lung cancer cell lines assessed by qRT-PCR are summarized in Table 2. As shown in Figure 1A, various amounts of CCL2 protein were produced in all of the lung cancer cell lines assessed, among which LK79, a SCLC cell line, produced notably high amounts of CCL2. The corresponding receptor, CCR2, was rarely expressed on the surface of both resting T cells or those activated using OKT-3/IL-2 (n = 5). Activated T cells were gated with CD69 positivity. A representative example among 5 cases is shown in Figure 1B. The levels of expression for resting and activated cells were: CD8, 0.83±0.72% and 0.75±0.51%, and CD4, 0.66±0.45% and 0.5±0.26% (mean± SD), respectively. Additionally, qRT-PCR revealed that the expression levels of *CCR2* mRNA in activated and resting T cells (n = 5) did not differ in either CD4^+^ or CD8^+^ T cells (data not shown). On the other hand, effector CD8^+^ T cells double-transfected to express HLA-A*2402-restricted and WT1_235–243_-specific-TCR successfully killed candidate human lung cancer cell lines in an HLA-A*2402-restricted manner (Fig. 1C and 1D). Taken together, the data suggested that the choice of the CCR2-CCL2 axis and the pairing of CTLs comprising effector cells gene-modified to express WT1-specific TCR with LK79 target cells that were sensitive to the cytocidal activity mediated by the transfected CTL was reasonably suitable for demonstrating the proof-of-concept of this study. Accordingly, these double-transfected effector CD8^+^ T cells paired with target LK79 cells were employed in the subsequent experiments.

**Table 2:** Production of chemokine mRNA by human lung cancer cell line cells.

Demographics of examined human lung cancer cell line										
	LC99A	LK79	RERF-LC-A1	LC11-18	PC-9	Sq-1	QG-56	LC65A	1–87	LK87
HLA-A*2402	+	+	+	+	+	−	−	−	−	−
#WT1mRNA	+	+	+	+	−	+	+	+	+	+

Production of chemokine mRNA by examined human lung cancer cell line										
Chemokine	LC99A	LK79	RERF-LC-A1	LC11-18	PC-9	Sq-1	QG-56	LC65A	1–87	LK87
CCL2	0.001	0.937	0.0004	0.505	0.001	0.002	0.005	0.330	0.021	0.413
CCL3	0	0	0	0	ND	ND	ND	ND	ND	ND
CCL4	0	0.001	0	0	ND	ND	ND	ND	ND	ND
CCL5	0.001	0.003	0.001	0.002	ND	ND	ND	ND	ND	ND
CCL8	0	0	0	0	ND	ND	ND	ND	ND	ND
CCL22	0	0	0	0	ND	ND	ND	ND	ND	ND
CXCL9	0	0	0	0	ND	ND	ND	ND	ND	ND
CXCL10	0	0.02	0	0	ND	ND	ND	ND	ND	ND
CXCL11	0	0.002	0	0	ND	ND	ND	ND	ND	ND
CXCL12	0	0	0	0.001	ND	ND	ND	ND	ND	ND
CXCL16	0.032	0.09	0.02	0.006	ND	ND	ND	ND	ND	ND
CX3CL1	0	0.11	0.006	0.034	ND	ND	ND	ND	ND	ND
*hHPRT1	1	1	1	1	1	1	1	1	1	1

# WT1mRNA positivity was reported in our previous paper (ref. No. 12).

* The expression level of each chemokine mRNA was corrected by reference to that of hHPRT1 mRNA.

ND indicates “not done”.

### 2. Functional validation of introduced CCR2

**Figure 2:**
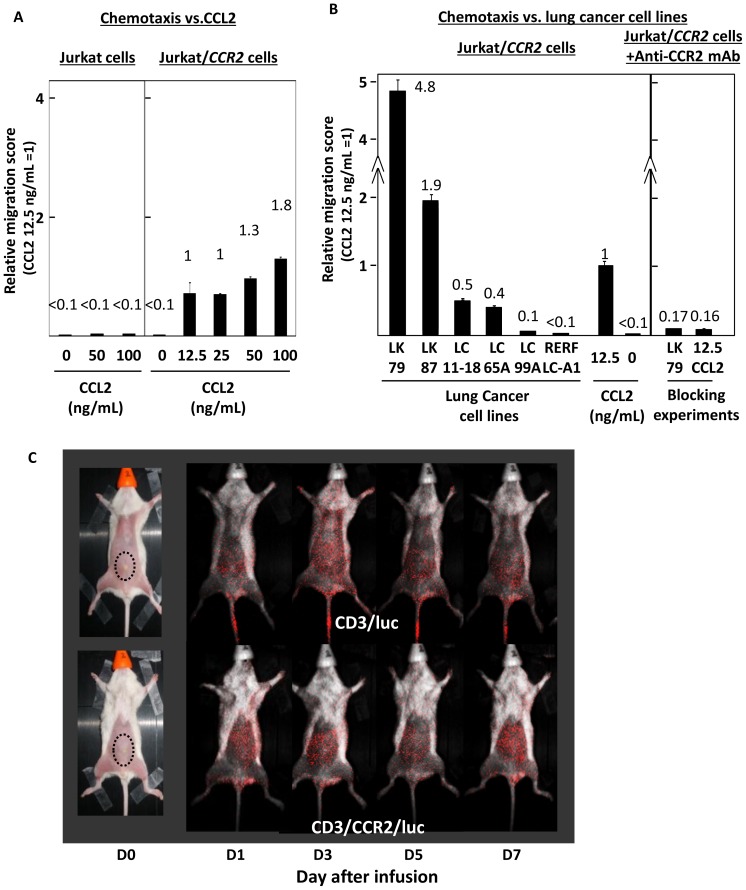
In vitro and in vivo functionality of introduced CCR2. (A) *CCR2*-transduced Jurkat cells, but not parental Jurkat cells, displayed dose-dependent transwell CCL2-chemotaxis. The numbers of cells migrating are shown relative to that at a CCL2 concentration of 12.5 ng/mL. Error bars represent SDs. (B) Similarly, the numbers of migrating *CCR2*-transduced Jurkat cells for each cancer cell line are shown. The migration of *CCR2*-transduced Jurkat cells was obviously inhibited by anti-CCR2 mAb, suggesting that the number of migrating cells was dependent on the level of CCL2 produced by each cell line (in Fig. 1A) and mediated by the introduced CCR2 on Jurkat cells. Error bars represent SDs. (C) CCL2-based target antigen-independent in vivo tumor trafficking towards inoculated LK79 cells mediated by *CCR2*-transfected CD3^+^ T cells was successfully demonstrated in a xenografted mouse model. Intravenously infused luciferase-labeled CD3^+^ T cells expressing CCR2 (CD3/CCR2/luc) (lower), but not those lacking CCR2 (CD3/luc) (upper), started to migrate towards LK79 cells immediately on day 1 after infusion, and their targeted migration was maintained throughout the observation period.

**Figure 3:**
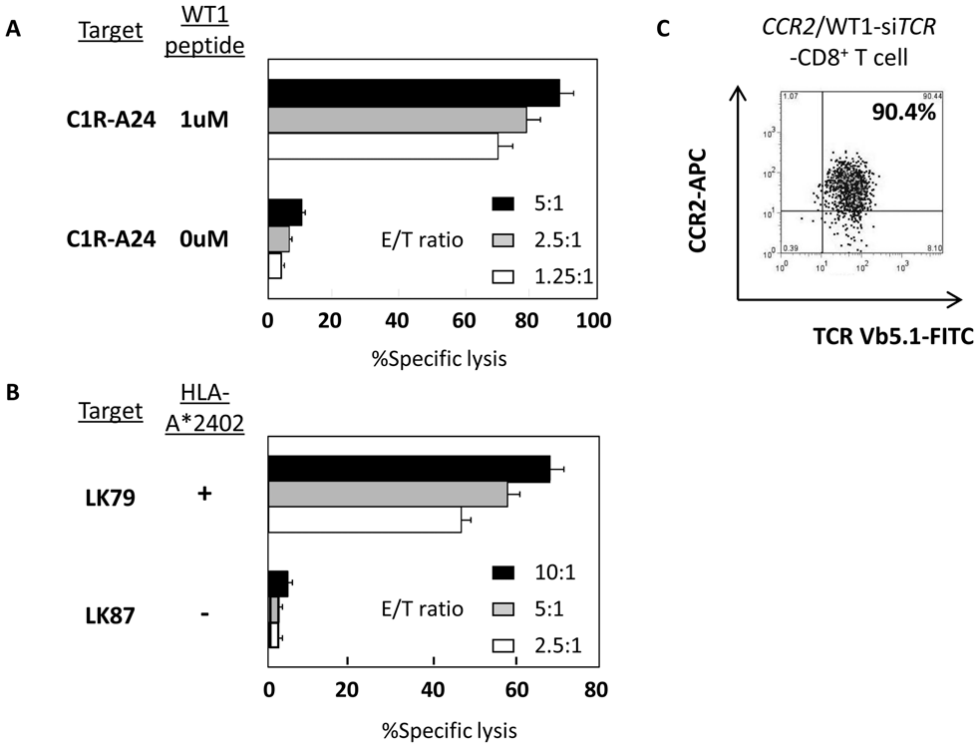
WT1-specific TCR-mediated cytocidal activity is not impaired by additional gene modification for expression of CCR2. (A) Using standard ^51^Cr release assay, these double-transfected effector cells sufficiently lysed 1 µM WT1 peptide-loaded, but not unloaded, C1R-A24 cells that expressed HLA-A*2402. E/T ratio indicates effector∶target ratio. Error bar indicates SDs. (B) These double-transfected effector cells also sufficiently lysed HLA-A*2402^+^ LK79 cells, but not LK87 cells lacking the HLA-A*2402 molecule used as a negative control. (C) Double-transfected CD8^+^ T cells successfully expressed both cell-surface CCR2 and Vβ5.1, the germ-line component of the WT1-specific TCR β chain. *CCR2*/WT1-si*TCR*-CD8^+^ T cells indicates double-transfected CD8^+^ T cells.

**Figure 4:**
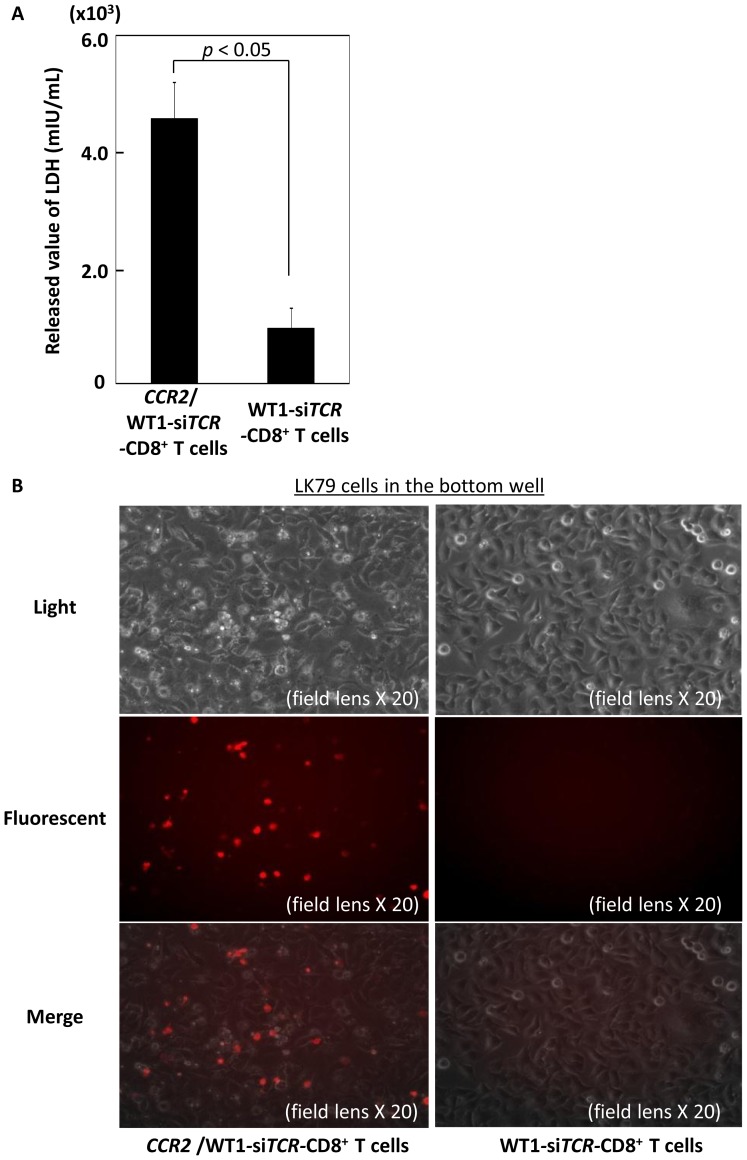
Transwell experiments for functional assessment of CD8^+^ T cells double-transfected to express both WT1-specific TCR and CCR2. (A) Double-transfected CD8^+^ effector cells (n = 3), but not WT1-si*TCR* single-transfected effector cells (n = 3), seeded in the upper well migrated to the bottom well more effectively, where they lysed seeded LK79 cells, as assessed in terms of the increase in the amount of LDH released from the disrupted LK79 cells. Error bars represent SDs. LDH indicates lactate dehydrogenase. (B) A representative set of light micrographs of three experiments, demonstrating a greater degree of LK79 cell destruction after treatment with double-transfected effector cells (top left), than with WT1-si*TCR* single-transfected effector cells (top right). Because CCR2 was genetically introduced along with a red fluorescent protein (DS red), only double gene-modified CCR2^+^ effector cells were detectable by red-colored labeling in the bottom well after migration (middle and bottom). In Figure 4A and 4B, *CCR2*/WT1-si*TCR*-CD8^+^ T cells indicates double-transfected CD8^+^ T cells, and WT1-si*TCR*-CD8^+^ T cells indicates WT1-si*TCR* single-transfected CD8^+^T cells.

Firstly, functional validation of introduced CCR2 was conducted using *CCR2*-transfected Jurkat cells (Jurkat/CCR2) in transwell experiments. Jurkat/CCR2, but not parental Jurkat cells, successfully displayed CCL2-mediated migration activity in a dose-dependent manner. Figure 2 shows the numbers of cells migrating relative to that at a CCL2 concentration of 12.5 ng/mL. Jurkat/CCR2 cells similarly displayed migration activity towards LK79, LK87, LC11-18, LC65A and LC99A cells seeded in the bottom wells according to the level of CCL2 produced by each cell line (Fig. 1A). Finally this chemotaxis mediated by Jurkat/CCR2 towards LK79 was completely inhibited by anti-CCR2 mAb (Fig. 2B). Next, using a xenografted NOG mouse model, tumor antigen-independent in vivo tumor trafficking mediated by *CCR2* and *luciferase* double-transfected normal CD3^+^ T cells was examined using bioluminescence imaging. One day after intravenous infusion, these luciferase-labeled CCR2-expressing CD3^+^ T cells started to accumulate at the site of inoculated LK79 cells in the anterior abdominal wall, whereas luciferase-labeled CD3^+^ T cells lacking CCR2 were dispersed throughout the entire body during the observation period (Fig. 2C). Next, validation of the function of double-transfected normal CD8^+^ T cells to express both WT1-specific TCR and CCR2 was carried out. First, we assessed whether the co-introduction of CCR2 impaired the WT1-specific cytocidal activity against lung cancer cells mediated by double-transfected effector CD8^+^ T cells. As shown in Figure 3A and 3B, WT1 peptide-responsive cytocidal activity and anti-lung cancer activity against HLA-A*2402^+^ LK79 cells (but not that against HLA-A*2402^−^ LK87 cells as negative control) mediated by these effector cells, being double-positive for Vβ5.1 and CCR2 (Fig. 3C), was not compromised relative to that mediated by WT1-specific *TCR* single-transfected CD8^+^ T cells (Fig. 1C). Next, the combined antitumor functionality against LK79 cells mediated by double-transfected CD8^+^ T cells, comprising both increased tumor trafficking activity and WT1-specific cytocidal activity, was examined using a modified transwell experiment. These double-transfected CD8^+^ T cells (n = 3), but not WT1-si*TCR* single-transfected CD8^+^ T cells (n = 3), in the upper well efficiently migrated into the bottom well where LK79 cells had been seeded and pre-incubated for 24 hours. Both double- and WT1-si*TCR* single-transfected effector cells loaded into the upper wells were equally 90–92% positive for Vβ5.1, and 70–72% of the double-transfectants expressed CCR2 (data not shown). Consequently, significantly (*p*<0.05) more LK79 cells were destroyed by migrated double-transfected CD8^+^ T cells than by WT1-si*TCR* single-transfectants, as represented by elevated levels of LDH in the culture supernatants (Fig. 4A). At the end of these transwell experiments, flow cytometric analysis revealed that 3.4 times more Vβ5.1-positive effector cells remained in the bottom well treated with double-transfected CD8^+^ T cells 7.91±0.51(×10^4^) for double-transfectants, and 2.35±0.13(×10^4^) for single-transfectants, respectively), supporting the results of the LDH assay. Microscopic examination confirmed a greater degree of destruction of LK79 cells in the bottom well after treatment with double-transfected effector cells, than with single-transfected cells. Furthermore, confocal microscopic examination demonstrated red-labeled migrated CCR2-expressing effector cells in the bottom well only after treatment with double gene-modified effector cells. A representative example among three experiments is shown in Figure 4B.

### 3. Co-introduced CCR2 augments in vivo anti-lung cancer reactivity mediated by double-transfected CD8^+^ T cells

**Figure 5:**
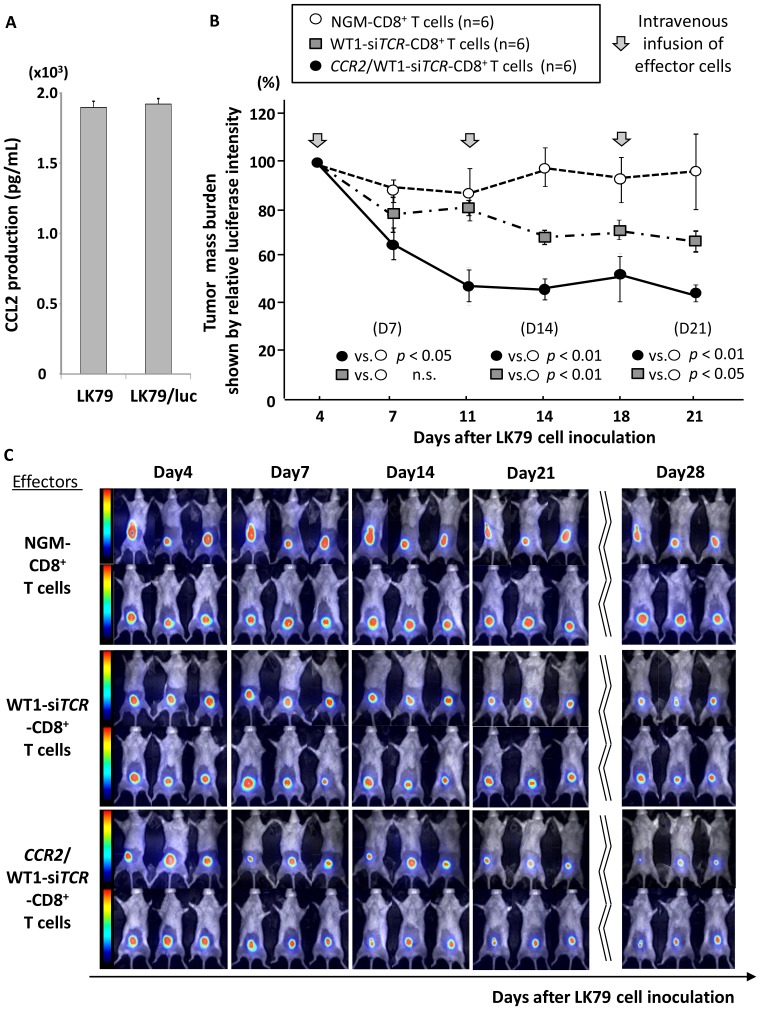
In vivo anti-lung cancer activity mediated by double-transfected effector cells in therapeutic xenografted mouse models. (A) The amount of CCL2 produced by *luciferase*-transfected LK79 cells (LK79/luc) was similar to that produced by the parent LK79 cells. (B) Nine-week-old NOG mice (n = 18) inoculated into the abdominal wall with 5×10^6^ LK79/luc cells were divided into 3 cohorts. On day 4, when each tumor mass had become palpable, three mice in each cohort started to receive weekly intravenous administration of 5×10^6^ double-transfected effector cells (cohort iii; black circles), WT1-si*TCR* single-transfected effector cells (cohort ii; gray square), or CD8^+^ T cells simply activated using OKT-3/IL-2 as a negative control (cohort i; clear circles), the effector cells all being generated from an identical donor. Intravenous administration was performed three times in total, and the relative mass burden was serially monitored on the basis of luciferase photon counts relative to those on day 4, before the start of therapeutic infusion. Double-transfected effector cells in cohort iii mice most effectively suppressed the growth of LK79/luc cells, notably in the immediate phase after therapeutic infusion (on day 7). In contrast, WT1-si*TCR* single-transfected effector cells gradually suppressed the growth of LK79/luc cells, being apparently dependent on time and the total number of effector cells infused. Effector cells that had been simply activated also displayed a marginal degree of tumor suppression, probably because of xenoreactivity. Error bars represent SDs. NGM-CD8^+^ T cells indicate CD8^+^ T cells simply activated using OKT-3/IL-2, expressing neither CCR2 nor WT1-specific TCR. (C) Serial bioluminescence images of mice in each cohort are shown. On day 28, 10 days after the last therapeutic infusion, durable growth suppression of LK79/luc cells was most evident in cohort iii mice that had received double-transfected effector cells. NGM-CD8^+^ T cells represent CD8^+^ T cells simply activated using OKT-3/IL-2, expressing neither CCR2 nor WT1-specific TCR.

Next, using a therapeutic xenografted mouse model, we examined in vivo anti-lung cancer reactivity mediated by infused effector cells double-transfected to express both WT1-specific TCR and CCR2. For this assay, we prepared *luciferase* gene-transduced LK79 cells (LK79/luc) that produced similar amounts of CCL2 to the parental LK79 cells (Fig. 5A). After 1 Gy of irradiation, cohorts of NOG mice (n = 6) were subcutaneously inoculated with 5×10^6^ LK79/luc cells in the anterior abdominal wall. Four days later, when the tumor mass had become palpable, these mice started to receive weekly intravenous administration of OKT-3/IL-2-activated, but not gene-modified, CD8^+^ T cells (cohort i), WT1-*siTCR* single-transfected CD8^+^ T cells (cohort ii) or *CCR2* and WT1-si*TCR* double-transfected CD8^+^ T cells (cohort iii), in a total of 3 infusions. As shown in Figure 5B, mice in cohort iii immediately displayed significant tumor suppression on day 3 after the first therapeutic infusion (*p*<0.01 for cohort iii vs. cohort i, *p*<0.05 for cohort iii vs. cohort ii), and thereafter the growth of LK79/luc was continuously suppressed until day 28, also with statistical significance (*p*<0.01 for both vs. cohort i and cohort ii). In contrast, WT1-si*TCR* single-transfected effector cells in cohort ii gradually suppressed the growth of LK79/luc cells. On day 14, the tumors in cohort ii mice first began to show a significant reduction in size relative to the mice in cohort i (p<0.01). Effector cells activated with only OKT-3/IL-2 in cohort i displayed a marginal tumor-suppressive effect, which was probably attributable to xenoreactivity. Serial bioluminescence images of the mice in each cohort are shown in Figure 5C. Co-introduction of functional CCR2 successfully enhanced in vivo anti-lung cancer reactivity mediated by infused double-transfected effector T cells, notably in the phase immediately after the start of therapeutic infusion, reflecting the increased tumor trafficking activity in response to CCL2.

### 4. Positive impact of the co-introduced CCR2-CCL2 axis on WT1-responsive TCR signaling in double-transfected effector cells

**Figure 6:**
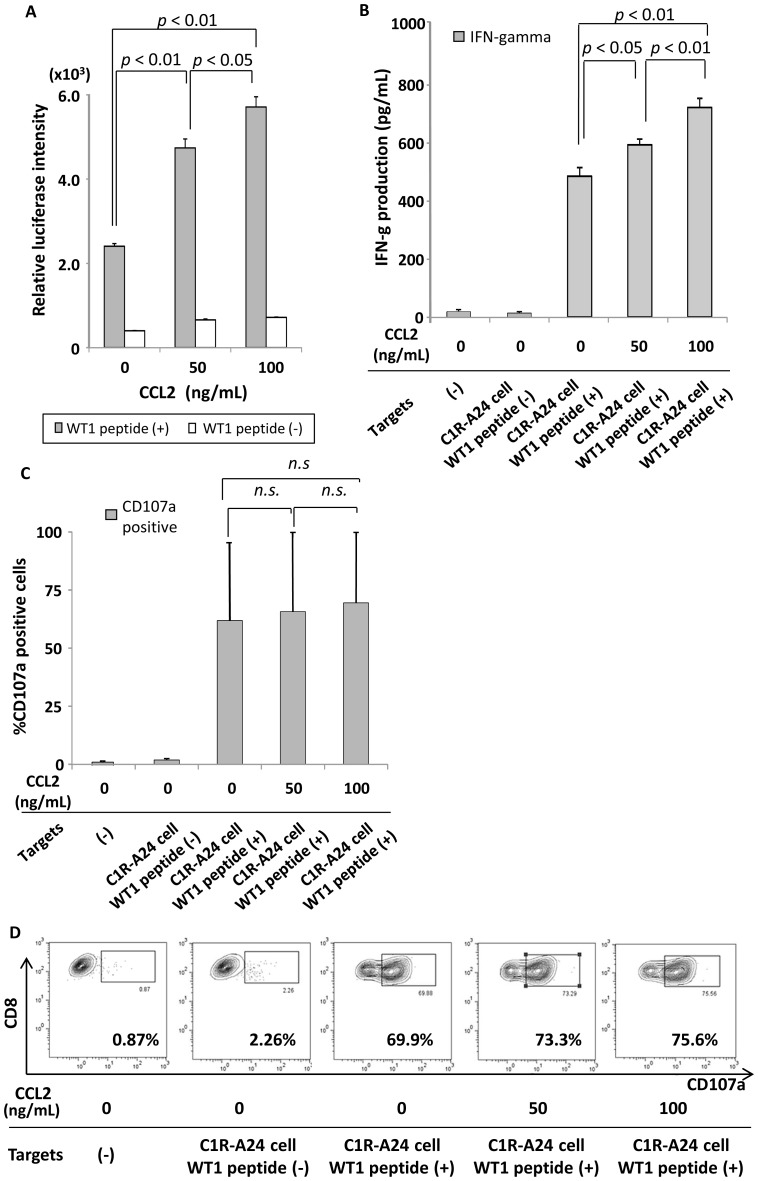
Positive cross-talk between CCR2 and WT1 epitope-responsive TCR signaling in double-transfected T cells. (A) In double-transfected Jurkat/MA/CD8α/luc cells, CCL2 ligation to introduced CCR2 significantly augmented WT1-responsive luciferase production triggered by stimulation with 20 µM WT1 peptide-loaded C1R-A24 cells in a dose-dependent manner. In the absence of WT1 peptide, CCL2 ligation induced a low level of luciferase production. (B) In normal peripheral CD8^+^ T cells (n = 3) double-transduced to express both CCR2 and WT1-specific TCR, CCL2 ligation significantly enhanced WT1-responsive IFN-γ production in response to stimulation with 1 µM WT1 peptide-loaded C1R-A24 cells in a dose-dependent manner. (C) Similarly treated double-transfected normal effector cells (n = 3) showed a trend to increase WT1-responsive cytotoxic degranulation, but not to a significant degree. (D) Representative data for CD107a expression from 3 cases examined are shown. (B), (C) and (D) illustrate that cellular outputs triggered by TCR ligation with the WT1 epitope/HLA complex were augmented in the presence of CCL2. n.s. indicates not significant.

Besides enhanced tumor trafficking activity, we attempted to examine the impact of the co-introduced CCR2-CCL2 axis on WT1-responsiveness itself in double-transfected effector cells. First, we generated double-transfected Jurkat/MA/CD8α/luc cells that expressed both WT1-specific TCR and CCR2. The magnitude of WT1-responsive TCR signaling in these cells became measurable using WT1 peptide-responsive luciferase production. WT1-si*TCR* and *CCR2* double-transfected Vβ5.1 and CCR2 double-positive Jurkat/MA/CD8α/luc cells were incubated with 20 µM heteroclitic WT1 peptide-loaded or unloaded C1R-A24 cells in several concentrations of CCL2, then subjected to luciferase assay. Experiments were carried out in triplicate and independently three times. It was found that CCL2 dose-dependently augmented the WT1 peptide-responsive luciferase production mediated by the double-transfected Jurkat/MA/CD8α/luc cells with statistical significance (Fig. 6A). Next, using similarly double-transfected normal CD8^+^ T cells obtained from 3 different individuals, being double-positive for HLA-A*2402/WT1_235–243_ tetramer and CCR2, we examined whether WT1 epitope-responsiveness could also be augmented in the presence of CCL2. All experiments were carried out in triplicate. It was found that the WT1 epitope-responsive IFN-γ production mediated by double-transfected CD8^+^ T cells was significantly upregulated in accordance with the concentration of CCL2 (Fig. 6B), while the WT1 epitope-responsive cytotoxic degranulation, as assessed in terms of increased cell-surface CD107a expression, also tended to increase, but not to a significant degree (Fig. 6C). Representative data for CD107a expression in three independent experiments are shown in Figure 6D. Collectively, the co-introduced CCR2-CCL2 axis potentiated not only tumor trafficking activity, but also WT1 epitope-responsiveness mediated by these double-transfected CTLs, resulting in enhanced anti-lung cancer functionality in vivo, notably in the phase immediately after therapeutic infusion.

## Discussion

In this study, using LK79 – a SCLC cell line – as a target, which produces high amounts of CCL2 (Table 2 and Fig. 1A), and effector CTLs double-transfected to express WT1-specific TCR and CCR2, we successfully demonstrated both the feasibility and advantages of targeting an optimal chemokine produced by tumor cells, to achieve successful adoptive therapy.

Targeting of an optimal chemokine produced by tumor cells or tumor-infiltrating immune cells to improve the antitumor efficacy mediated by tumor-reactive T cells was first demonstrated in vitro by Kershaw et al. [15]. Subsequently, a series of preclinical studies showed that the increased antitumor functionality mediated by these effector cells was mainly attributable to enhanced tumor trafficking activity caused by the introduced chemokine receptor [16]–[19]. An early clinical trial using ex vivo-expanded and radiolabeled TILs for treatment of patients with advanced melanoma had already demonstrated that localization of infused TILs was an important determinant of clinical efficacy [27]. More recently, using biopsied tissues from melanoma patients, Harlin et al. confirmed the clinical importance of the chemokine production pattern in tumor tissues as a critical determinant of the effectiveness of antitumor immunity [28]. Although targeting of appropriate chemokines produced by tumor cells or infiltrating elements within the tumor microenvironment has become an attractive option for increasing the numbers of tumor-infiltrating effector cells, this approach is still at the preclinical stage. The fundamental issue of which chosen chemokine would be optimal for use against a wide range of tumors still remains to be explored.

CCL2, a CC chemokine that stimulates CCR2, its corresponding receptor expressed on T cells [29], NK cells [30] and γδ T cells [31], is reported to be expressed by various types of cancer cells including those of glioma [17], melanoma [28], and cancers of the breast [32], prostate [33], colon [34] and lung [35]. Our method used for retroviral transduction of the WT1-si*TCR* gene into normal CD8^+^ T cells involves stimulation of T cells using OKT-3/IL-2 in a RetroNectin-coated plate [6]. Whenever we transduced the WT1-si*TCR* gene, CCR2 was rarely expressed on activated CD8^+^ T cells (Fig. 1B). Moreover, positivity for cell-surface CCR2 could only be achieved by *CCR2* gene-transfection. Consequently, the *CCR2*-transfectants acquired sufficient CCL2-responsiveness both in vitro and in vivo (Figs. 2 and 4), thus confirming the therapeutic efficacy and feasibility of introducing the CCR2-CCL2 axis into tumoricidal T cells. On the other hand, a previous report has indicated that CCR2 expression in activated T cells was functionally positive [36]. This discrepancy was likely due to the difference in the method used for stimulating T cells, as Craddock et al. have previously discussed [17].

On the other hand, the suppressive role of CCL2 in antitumor immunity has also been intensively studied. CCL2 produced in the tumor microenvironment is known to recruit tumor-associated macrophages (TAMs) [37], which are known to support the growth of neuroblastoma cells via production of IL-6 [38], to suppress antitumor immunity in cancer-bearing hosts via IL-10 production [39] and recruitment of regulatory T cells [40], and to promote tumor-supportive angiogenesis [41]. Furthermore, TAM itself produces CCL2 and thus amplifies this circuit [37]. However, even though CCL2 in the tumor microenvironment is diversely implicated in the suppression of host antitumor immunity, abundant production of CCL2 by tumor cells or infiltrating immune cells might be advantageous for efficient localized recruitment of therapeutically infused tumoricidal CCR2-expressing T cells into tumor tissues. As well as increased tumor trafficking activity, we demonstrated that the co-introduced CCR2-CCL2 axis might also be advantageous for potentiating target-responsive cytocidal activity, i.e., WT1-specific TCR-mediated anti-lung cancer reactivity. Double-transfected Jurkat/MA/CD8α/luc cells displayed increased production of luciferase triggered by WT1 peptide-responsive TCR signaling, and this effect was dependent on the concentration of CCL2. Even without WT1 peptide stimulation, CCL2 stimulated the cells to produce a small amount of luciferase, suggesting that influx of calcium into the double-transfected Jurkat/MA/CD8α/luc cells triggered by CCL2 ligation might, to some extent, directly stimulate the NFAT pathway shared by WT1-responsive TCR signaling (Fig. 6A). Finally, in the presence of CCL2, augmentation of both WT1 epitope-responsive IFN-γ production and CD107a expression was mediated by double-transfected normal CD8^+^ T cells stimulated using WT1 peptide-loaded C1R-A24 cells (Fig. 6B, 6C and 6D). With regard to the interplay between chemokine receptors and TCR in the same effector T cell, the role of the CXCR4-SDF-α axis in T cell activation [42], [43], [44] and that of the CCR7-CCL19/CCL21 axis in T-cell homing to lymph nodes [45] have been studied in detail. In our system, although details of the mechanism still remain undetermined, it can be suggested that after CCR2-CCL2 axis-mediated migration into tumor tissues, CCL2 in the tumor microenvironment may strengthen WT1 epitope-responsive cytocidal activity against LK79 cells mediated by infused double-transfected effector CD8^+^ T cells in vivo.

In our previous study, we demonstrated the therapeutic potential of intravenously infused WT1-specific CTL clone cells (TAK-1) against human lung cancer cells in vivo [12]. In the present study, we demonstrated the WT1-specific anti-lung cancer effect mediated by CD8^+^ T cells genetically engineered to express WT1-specific TCR originating from TAK-1 [13]. Furthermore, we successfully provided experimental evidence that the suppression of tumor growth mediated by *CCR2* and WT1-si*TCR* double-transfected effector T cells was more effective than that mediated by WT1-si*TCR* single-transfected cells (Fig. 5B and 5C). In human lung cancers, expression of both *WT1* mRNA and WT1 protein has been demonstrated in tumor cells, suggesting the therapeutic potential of WT1 as a target of anti-lung cancer immunotherapy [10], [46]. Indeed Oka et al. have reported clinical responses in lung cancer patients after WT1 peptide vaccination [47].

Non-malignant cells including fibroblasts, endothelial cells, smooth muscle and microglial and astrocytic cells, also produce CCL2 under physiological or inflammatory conditions [48]. These cells might cause CCR2-introduced effector cells to become dispersed into those tissues, thus blunting any therapeutic effect or causing unintended adverse events. However, because the lung is the first-pass organ for intravenously infused effector cells, when intending to treat lung cancer, CCL2 at tumor sites in the lung field would preferentially attract such infused effector cells to a much greater extent than CCL2 expressed physiologically in other peripheral tissues.

In the present study, we have demonstrated both the feasibility and advantages of CD8^+^ T cells double-transfected to express WT1-specific TCR and CCR2 for the treatment of at least some human lung cancers. Similar studies using T cells genetically redirected to express CCR2 and CARs for the treatment of neuroblastoma [17] and malignant mesothelioma [19] also appear to support the idea that the CCR2-CCL2 axis is a rational choice for use in redirected T cell-based anticancer adoptive immunotherapy. Further studies are needed to assess the clinical potential of this strategy.
